# Transcatheter thrombolytic therapy for symptomatic thrombo-occlusion of inferior vena cava filter

**DOI:** 10.3892/etm.2012.843

**Published:** 2012-11-30

**Authors:** LIANG XIAO, JING SHEN, JIA-JIE TONG, ZHE ZHANG, XIAO-LIN MU, ZHENG-JIA YI, SHUO BAI, KE XU

**Affiliations:** 1 Department of Radiology, The First Hospital of China Medical University, Liaoning, Shenyang 110001;; 2 Department of Intervention, Shanxi Provincial People’s Hospital, Shanxi, Taiyuan 030012;; 3 Medical Imaging Center, Hebei Provincial People’s Hospital, Shijiazhuang, Hebei 050051, P.R. China

**Keywords:** thrombosis, filter, inferior vena cava, thrombolysis, efficiency

## Abstract

Thrombus within an inferior vena cava (IVC) filter reduces filter patency and venous return from the lower extremities, and may progress to complete IVC occlusion. The clinical experiences and outcomes of transcatheter thrombolytic therapy for symptomatic IVC thrombosis following filter implantation have not been widely reported. The aim of the current study was to evaluate the efficiency and safety of trans-catheter thrombolysis for the treatment of symptomatic IVC thrombosis in patients with implanted IVC filters. Transcatheter thrombolysis was used to treat 5 patients with thrombosis of the filter-bearing IVC causing symptoms in 10 limbs from October 2005 to September 2010. The patients were implanted with a second IVC filter through the right internal jugular vein, followed by recanalization of the occluded IVC and intravenous transcatheter thrombolysis. The IVC filters were retrieved through the femoral or right internal jugular vein after the thrombus had dissolved. Technical and clinical outcome, complications and postoperative pulmonary embolism were monitored. A total of 5 filters were implanted and 6 filters were retrieved later. Technically and clinically successful recanalization and thrombolysis were achieved in 5 of 5 patients and 10 of 10 symptomatic limbs. The median thrombolysis period was 13 days (range, 8–14 days). The median dwell time for the filters that were removed was 50.5 days (range, 14–73 days). No major bleeding occurred during the current study. During clinical follow-up, no clinically detectable pulmonary embolism was observed. Endovascular recanalization and transcatheter thrombolysis of IVC thrombosis are efficient, feasible and safe in the presence of an IVC filter.

## Introduction

Implantation of an inferior vena cava (IVC) filter is safe and effective in the prevention or reduction of fatal pulmonary embolism (PE). However, there are risks associated with long-term implantation of these filters, including IVC occlusion, thrombosis and the recurrence of deep venous thrombosis (1–3).

Thrombus within an IVC filter reduces filter patency and venous return from the lower extremities, and may progress to complete IVC occlusion. Depending on collateral formation and the extent of venous involvement and valvular damage, the long-term sequelae of IVC thrombosis may range from mild ambulatory lower extremity swelling to incapacitating edema at rest, venous claudication and/or venous ulcers. In addition, filter thrombosis may result in recurrent PE secondary to thrombus propagation above the filter (4,5). Renal failure secondary to IVC filter thrombus propagation into the renal veins has been reported (6–8). If untreated or inadequately treated, this condition may cause debilitating lower extremity pain and swelling, back pain, weakness and venous stasis ulceration. Conservative therapy (pneumatic and elastic compression, leg elevation and/or anticoagulation) alone is usually inadequate to relieve symptoms, and venous bypass surgery has been the only option in the most severe cases and has had limited efficacy and applicability (9). Transcatheter thrombolytic therapy has demonstrated short-term effectiveness in the treatment of patients with iliofemoral deep vein thrombosis (DVT) and limited numbers of patients with IVC thrombosis (10–14).

We have employed transcatheter thrombolysis to treat patients who have developed symptomatic IVC thrombosis following filter implantation elsewhere and have been referred to the Radiology department of the First hospital of China Medical University. In the present study, we describe the clinical experiences and outcomes of five cases complicated by acutely/subacutely symptomatic IVC thrombosis following filter implantation.

## Patients and methods

### Patients

Institutional review board approval was obtained from the ethics committee of the First Hospital of China Medical University for this study. Between October 2005 and September 2010, 5 patients were referred to our department with symptomatic IVC thrombosis following filter implantation. The patients comprised 5 males, with a mean age of 34.2 years (range, 17–54 years). The onset time was from 2 to 30 days. Symptoms included bilateral lower limb swelling (n=5), pain (n=1), cyanosis (n=2) or pallescence (n=3) and rising (n=3) skin temperature. Patient demographics, symptoms, indication for filter placement, filter type, the interval between filter placement and symptoms emerging and the interval between symptoms emerging and transcatheter thrombolysis for each patient are recorded in Table I. Interventional surgery was performed after informed consent was obtained from the patient.

### Filter implantation and IVC transcatheter thrombolysis

Through the right jugular vein approach, a 5-Fr pig-tail catheter was placed into the IVC above the filter. An anteroposterior cavogram was performed to ensure the location of the previous IVC filter, the extent of IVC thrombosis, the fluency of the bilateral renal veins and the diameter of the suprarenal IVC. After confirming that the suprarenal IVC was free of thrombus, an 8.5-Fr sheath was inserted into the IVC. An OptEase filter (Cordis Corp., Miami Lakes, FL, USA) or a Günther Tulip filter (GTF; Vena Cava MReye Filter set; William Cook Europe, Bjaeverskov, Denmark) was placed into the sheath and moved forward until the distal end of the filter reached 1–2 cm above the level of the renal vein confluence. The sheath was slowly withdrawn, allowing the filter to enter the caval lumen and unfold. After ensuring that the filter was suitably located and free of tilt, the retrieval hook of the GTF was released.

After excluding PE by an anteroposterior pulmonary arteriogram, a 5-Fr curved catheter over a 0.035-inch hydrophilous guide wire was placed into the IVC above the occluded filter. The guide wire was pushed forwards and rotated when its tip met resistance from the thrombus. In acutely IVC thrombotic patients, the guide wire easily entered the thrombus. In subacute patients, the guide wire entered the thrombus with assistance from the catheter. The catheter then entered the thrombus over the guide wire. This process was repeated until the catheter tip entered the distal vein lumen without thrombus. A venogram was performed to ensure the extent of the thrombus and collateral veins. Then, a thrombolytic catheter (UniFuse; AngioDynamics, Queensbury, NY, USA) with a 20 or 30 cm-length side-hole was placed into the thrombus. 500,000 IU urokinase was infused for 2 h twice every day through the thrombolytic catheter. Systemic anticoagulant therapy was administered by intravenously injecting 50 IU/kg heparin every 6 h. A cavogram and lower extremity venogram were performed every 3–4 days during thrombolysis therapy. The location of thrombolytic catheter was adjusted according to the extent of the residual thrombus.

### Filter retrieval

When the symptoms of the bilateral lower extremities were partly or completely relieved, and no or minimal residual thrombus remained in the IVC and bilateral iliofemoral veins, the thrombolytic therapy was terminated and the IVC filter was retrieved within a limited period.

Through the right jugular vein approach for retrieval of the GTF, a 5-Fr pig-tail catheter was placed into the IVC above the bifurcation. An anteroposterior cavogram was performed to ensure the tilt angle of the GTF and the relationship between the apical retrieval hook and the IVC wall, and to ensure that the filter was free of thrombus or had captured <8 mm thrombus. A 12-Fr sheath was then inserted into the IVC near the filter. A 15-mm goose-neck snare (Amplatz Goose Neck; ev3 Inc., Plymouth, MN, USA) was passed through the sheath. The retrieval hook of the filter was engaged with the snare, the snare was closed and the filter was sheathed.

Through the femoral vein approach for retrieval of the OptEase filter, a pig-tail catheter was placed into the iliac vein. After ensuring that the filter was free of thrombus or had captured <8 mm thrombus by an anteroposterior cavogram, a 12-Fr sheath was inserted into the IVC near the filter. The snare was passed through the sheath. The retrieval hook of the filter was engaged with the snare and the filter was sheathed. If repeated attempts, including operations performed under the Valsalva maneuver, did not engage the retrieval hook, it was necessary to use a catheter-directed technique. In the catheter-directed technique, the snare trapped the head end of a 5-Fr curved catheter. The catheter was then pushed forward. When the curved portion of the catheter entered the filter cone, the catheter was rotated and twisted with the strut of the filter cone. The snare was loosened slightly and pushed slowly along the catheter under the Valsalva maneuver. When the snare had trapped the filter cone, the catheter was drawn back. The snare then encircled the retrieval hook, the sheath was pushed forward and the filter was sheathed.

When the filter was enclosed by the sheath, the filter was retracted and removed. A repeat cavogram and pulmonary arteriogram were obtained following retrieval to inspect for complications. The technical and clinical outcome, complications and postoperative PE were monitored. All patients accepted long-term anticoagulation treatment by oral warfarin following the retrieval procedure. The patients were examined by vascular ultrasound 6 months after the surgery.

## Results

**Figure 1.**
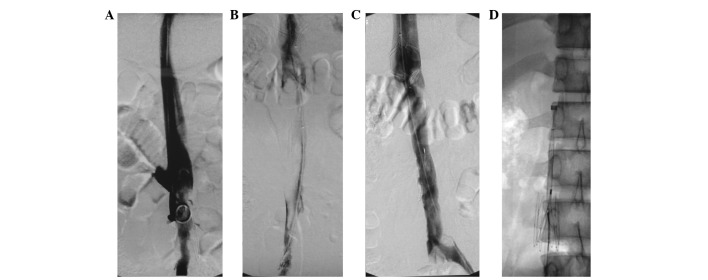
Case 1: Bilateral lower limb swelling and pain of 2 days’ duration, 7 days after filter implantation. (A) Occlusion of the infrarenal IVC and Vena Tech filter in the thrombus were displayed by cavogram. (B) Following implantation of an OptEase filter in the suprarenal IVC and recanalization using a guide wire, the venogram displayed a large thrombus in the IVC. (C) After 14 days of thrombolytic therapy, the amount of residual thrombus in the IVC was <10%. (D) The OptEase filter was retrieved through a femoral vein approach. IVC, inferior vena cava.

**Figure 2.**
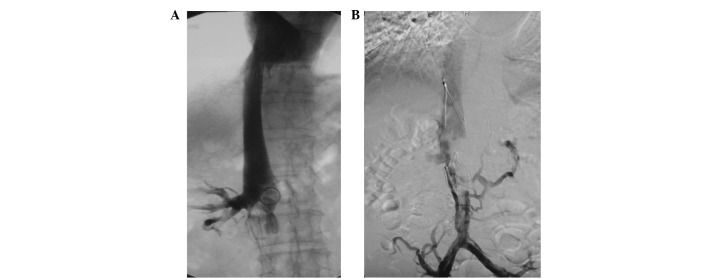
Case 4: Bilateral lower limb swelling of 30 days’ duration, 11 months after filter implantation. (A) Occlusion of the infrarenal IVC and OptEase filter in the thrombus were displayed by cavogram. (B) After 14 days of thrombolytic therapy, the residual thrombus in the IVC was >50% and the bilateral lower limb swelling was relieved significantly. IVC, inferior vena cava.

Five retrievable filters, including 4 GTFs and 1 OptEase filter, were successfully implanted in 5 patients. The median duration of the filter implantation surgery was 2.0 min (range, 1–3 min). Technically and clinically successful recanalization and thrombolysis were achieved in 5 of 5 patients and 10 of 10 symptomatic limbs. In 4 patients, the residual thrombus in the IVC was <10% (Fig. 1). The residual thrombus in the IVC was >50% in one patient (Fig. 2). The median duration of the IVC recanalization surgery was 5 min (range, 3–15 min). The median thrombolysis period was 13 days (range, 8–14 days). No major bleeding occurred during the study.

Six retrievable filters, including GTF (n=4) and OptEase filters (n=2), were finally retrieved, a success rate of 100% (6/6). In case 2, the OptEase filter in the thrombus was successfully retrieved using the catheter-directed technique when the thrombolytic therapy had been administered for 4 days and the residual thrombus in the IVC and OptEase filter was still large. The median dwell time for the filters that were removed was 50.5 days (range, 14–73 days). The median duration of the fluoroscopic retrieval surgery was 5.5 min (range, 2–26 min). No procedure-related complications occurred. During clinical follow-up, no clinically detectable PE or lower extremity swelling were observed. The type of second filter, dose of urokinase, course of transcatheter thrombolysis, IVC thrombus residue rate and dwell time of the retrieved filter for each patient are recorded in Table II.

## Discussion

**Figure 3.**
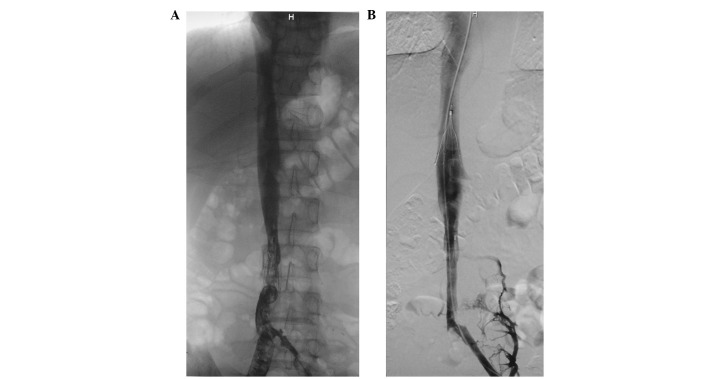
Case 3: Bilateral lower limb swelling of 5 days’ duration, 25 months after filter implantation. (A) After recanalization by guide wire, the cavogram displayed a large thrombus in the infrarenal IVC and a Vena Tech filter in the thrombus. (B) After 11 days of thrombolytic therapy, the residual thrombus in the IVC was <10%. IVC, inferior vena cava.

Complications of inferior vena cava filters include thrombosis of the IVC, caval occlusion, recurrent DVT, recurrent pulmonary embolus, filter migration, caval and aortic perforation and struts fracture (15,16). Thrombosis of the IVC is a potentially catastrophic complication of caval filter placement, and its reported incidence ranges from 0.8 to 25%, depending on filter type, indication of filter implantation and anticoagulant therapy (5,17–21). Due to the draining of lateral veins, some patients with thrombosis of the IVC following filter implantation may not suffer symptoms such as swelling and pain in both lower extremities and the rate of thrombosis of the IVC following filter implantation may be significantly higher than that of symptomatic IVC thrombosis. A comparative analysis revealed that there was a significantly higher incidence of symptomatic IVC thrombosis with the use of the Bird’s Nest filters (14.6%) compared with the stainless steel Greenfield filters (0%), titanium Greenfield filters with a modified hook (3.6%) or Vena Tech filters (4%; P<0.05) (17). In a clinical study of 400 cases, Nazzal *et al* reported that in the group of patients who had hypercoagulable conditions, the incidence of IVC thrombosis was higher with TrapEase filters compared with all other filters as a group (P<0.05) (21). Crochet *et al*(22) studied 142 patients with implanted Vena Tech filters and found a caval patency rate of 80% at a 9-year follow-up. Patients who received filters due to anticoagulation failure had a significantly higher (P=0.016) rate of filter occlusion (64.8%) (22). Tardy *et al*(5) reported that in 30 consecutive cases with symptomatic IVC filter thrombosis, 25 patients did not receive anticoagulant at the time of the diagnosis and none of the other five received adjusted anticoagulation. In the current study, 5 patients suffered symptomatic IVC thrombosis with the use of Vena Tech filters (n=2; Fig. 3) and OptEase filters (n=3). In cases 1 and 2, the anticoagulant therapy following filter implantation was inadequate, the symptoms worsened gradually, and the patients were referred to our hospital after being diagnosed with IVC thrombosis. In cases 3–5, the patients were free from clinical symptoms during the anticoagulant therapy and the swelling of lower extremities emerged 1 year, 3 months and 4 months after the cessation of anticoagulant therapy, respectively. The mechanisms of thrombosis of the IVC following filter implantation remain obscure. The authors consider that it is related to the hemodynamic changes in the IVC following filter implantation, captured thrombus in the filter and local thrombosis at the site where the filter struts contact the IVC wall due to the stimulus of the filter’s radial force, and anticoagulant therapy after filter implantation may reduce the extent of thrombosis in these conditions. In addition, filter retrieval when the risk of PE has decreased may be a prophylactic method for IVC thrombosis.

The therapeutic methods for symptomatic IVC thrombosis following filter implantation include anticoagulant therapy, systemic thrombolysis, catheter-directed thrombolysis, mechanical thrombectomy, balloon dilation, stent placement and filter retrieval (5,7,23–31).

Anticoagulation therapy is largely ineffective in relieving the symptoms of thrombosed IVC in the lower extremities (32). Thrombolytic therapy is of value in acute thrombosis. Bihorac and Kitchens reported that systemic thrombolytic therapy successfully treated acute kidney injury secondary to thrombosis of a suprarenal IVC filter (23). Infusion of tissue plasminogen activator (tPA) through a thrombolytic catheter has successfully treated a patient with bilateral renal vein thrombosis due to IVC filter migration and thrombosis (7). Angle *et al* reported that thrombolysis for symptomatic IVC thrombosis was successful in 7 of 8 (88%) patients with no or minimal residual thrombus using local catheter-directed infusion of urokinase and the 7 patients had no lower extremity swelling after the procedure (24). In the current study, after infusion of urokinase through a thrombolytic catheter, the residual thrombus in the IVC was <10% in 4 of 5 patients with a thrombo-occlusive IVC filter, the blood flow in the IVC was recovered in all 5 patients, and swelling of the lower extremity disappeared or was relieved in all 10 limbs.

Bleeding is a severe complication of thrombolytic therapy. Patients who have an anticoagulant contraindication or a bleeding tendency are not suitable for treatment with thrombolytic drugs. Under such conditions, percutaneous mechanical thrombectomy has been used as an alternative therapy which may result in rapid symptomatic relief (25). In addition, combined catheter-directed thrombolysis and mechanical thrombectomy may shorten the course of treatment and decrease the dose of thrombolytic agent for acute IVC thrombosis (26).

Treatment with suction thrombectomy and thrombolysis is usually ineffective for chronic thrombosis. To maintain the fluency of the IVC, balloon dilation and stent implantation have been performed following the recanalization of chronic IVC thrombosis. Joshi *et al* reported that following thrombolysis, thrombectomy and percutaneous removal of the filter did not succeed and an expandable metallic Gianturco Z stent (Cook, Bloomington, IN, USA) was used to extract the TrapEase filter from the vessel lumen (27). In 25 patients with an obstructed IVC filter, following recanalization by a guide wire and balloon dilation, the filter was markedly displaced sidewise or remodeled (28). Following the implantation of a stent across the filter and re-dilation, the lumen of the IVC was recovered. Stenting maneuvers through a previous IVC filter have been safely performed with no tearing of the IVC, no clinical bleeding or abdominal symptoms and no pulmonary embolism (28–30).

**Figure 4.**
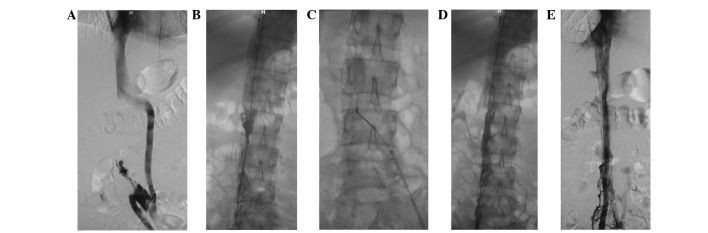
Case 2: Bilateral lower limb swelling of 3 days’ duration, 10 days after filter implantation. (A) Occlusion of the infrarenal IVC, OptEase filter in the thrombus and collateral vein were displayed by anterograde cavogram through the left iliac vein. (B) Following the implantation of a Günther Tulip filter in the suprarenal IVC and recanalization by guide wire, the venogram displayed a large thrombus in the IVC. (C) After 4 days of thrombolytic therapy, the OptEase filter was retrieved through a femoral vein approach. (D) After retrieval of the OptEase filter, the cavogram displayed thrombus in the IVC and no tearing of the IVC. (E) After another 4 days of thrombolytic therapy, the residual thrombus in the IVC was <10%. IVC, inferior vena cava.

Retrieval of a thrombo-occlusive IVC filter may not only remove a thrombosis-evoking factor, but also help to increase the efficacy of other therapies for previous thrombus. The retrieval surgeries of thrombotic IVC filters are usually complicated and time-consuming. In a clinical study, Kuo *et al* reported the experience of filter retrieval for treating symptomatic filter-related IVC stenosis and thrombotic complications (31). In the current study, an OptEase filter with inner thrombosis was successfully retrieved during transcatheter thrombolytic therapy. The thrombolytic therapy was continued for another 4 days until the residual thrombus in the IVC and iliofemoral veins was <10% (Fig. 4). The course of thrombolytic therapy in this patient was the shortest in our study.

Endovascular recanalization and transcatheter thrombolysis of IVC thrombosis are efficient, feasible and safe, even in the presence of an IVC filter. The prompt diagnosis and interventional therapy are likely to improve the prognosis of symptomatic IVC thrombosis following filter implantation.

## Figures and Tables

**Table I.** Demographic and clinical characteristics of the patients.

Case no.	Gender/age (years)	Type of filter	Indication for filter implantation	Interval between filter implantation and symptoms emerging	Symptoms of IVC occlusion	Interval between symptoms emerging and thrombolysis
1	M/17	Vena Tech	Left DVT	5 days	Swelling and pain in both LE	2 days
2	M/24	OptEase	Right DVT	7 days	Swelling in both LE	3 days
3	M/28	Vena Tech	Left DVT	25 months	Swelling in both LE	5 days
4	M/54	OptEase	Left DVT	10 months	Swelling in both LE	30 days
5	M/48	OptEase	Right DVT	21 months	Swelling in both LE	7 days

DVT, deep vein thrombosis; LE, lower extremities; IVC, inferior vena cava.

**Table II.** Interventional therapy of the patients.

Case no.	First filter	Second filter	Dose of urokinase (U/bid)	Course of treatment (days)	IVC thrombus residue (%)	Dwell time (days)
1	Vena Tech	OptEase	500,000	14	<10	14
2	OptEase	Tulip	500,000	8	<10	52 (14)^a^
3	Vena Tech	Tulip	500,000	11	<10	49
4	OptEase	Tulip	500,000	14	>50	73
5	OptEase	Tulip	500,000	13	<10	62

a Dwell time of the Tulip filter was 52 days and the dwell time of the OptEase filter was 14 days. IVC, inferior vena cava.
